# *Micromyzus platycerii* sp. n. (Hemiptera, Aphididae) – a new fern-feeding aphid species from Thailand

**DOI:** 10.3897/zookeys.456.8598

**Published:** 2014-11-21

**Authors:** Ewa Mróz, Łukasz Depa, Taksin Artchawakom, Jacek Gorczyca

**Affiliations:** 1 Department of Zoology, University of Silesia, Bankowa 9, 40-007 Katowice, Poland; 2 Sakaerat Environmental Research Station, Nakhon Ratchasima Province, Wang Nam Khieo District, Thailand

**Keywords:** Sakaerat Research Station, *Platycerium*, Macrosiphini

## Abstract

A new fern-feeding aphid species, *Micromyzus platycerii*, collected in Sakaerat Research Station in Thailand, is described.

## Introduction

There are approximately 66 species of fern-feeding aphids worldwide, belonging to 18 genera (Blackman and Eastop 2006). A few of them are polyphagous species more commonly found on angiosperms, but most of them are specialized on ferns. Most of these specialized genera occur in tropical and subtropical regions and their geographical ranges overlap considerably.

Among them are two very similar and probably related fern-feeding aphid genera: *Micromyzella* Eastop and *Micromyzus* van der Goot. *Micromyzella* comprises around 12 species, inhabiting mainly south-east Africa but also the Philippines and Indonesia (Remaudière and Autrique 1985). Its main morphological characteristics are 2–3 setae on first tarsal segments (including 1 sensory, peg-like seta) and, in alate females, darkly colored wing veins with the radial sector moderately curved (Blackman and Eastop 2006). *Micromyzus* is similar, but usually with 4 setae on the first tarsal segments (including 2 sensory peg-like setae) and, in alate females, heavily bordered wing veins and a strongly curved radial sector. It comprises about 10 species, distributed mainly in south-east Asia.

The Sakaerat Environmental Research Station (SERS) is situated on the edge of Thailand’s Khorat Plateau approximately 300 km north-east of Bangkok (SERS 2014). It was created in 1977, as a site for research on dry evergreen and dry dipterocarp tropical forest (Trisurat 2010). Other vegetation types in the biosphere reserve include bamboo forests, forest plantations and grasslands. During the field studies in SERS in January 2014, samples of fern-feeding aphids were collected. Here we describe a new species of fern-feeding aphid belonging to the genus *Micromyzus*, collected recently in Sakaerat Environmental Research Station.

## Material and methods

### Type material

no. UŚ 28.01.A/2014, 1 apterous viviparous female, labeled: Holotype; 1 apterous and 1 alate viviparous females, labeled: Paratypes; leg. J. Gorczyca

no. UŚ 28.01.B.1/2014, 4 apterous viviparous females, labeled: Pataypes; leg. J. Gorczyca

no. UŚ 28.01.B.2/2014, 1 alate viviparous female, labeled: Paratype; leg. J. Gorczyca

Collection site: 14°29’50.4”N; 101°55’20.9”E.

Collection data: Sakaerat Research Station, Thailand; 28.01.2014;

All slides are deposited in the collection of the Department of Zoology, University of Silesia (UŚ).

Measurements and diagnostic, morphological features follow Takahashi 1925, Miyazaki 1968, Noordam 2004 and Blackman and Eastop 2006.

## Descriptions

### Micromyzus platycerii

Taxon classificationAnimaliaHemipteraAphididae

Mróz & Depa sp. n.

http://zoobank.org/2B599791-2ED2-4D4B-AC09-1A2E8A1AAB11

#### Etymology.

Named after its host plant, from which it was collected: *Platycerium coronarium* (Konig) Desv.

#### Diagnosis.

The species belongs to the genus *Micromyzus*, because its alate female has dark-bordered wing veins and strongly curved radial sector. The new species differs from other representatives of the genus *Micromyzus* (including *Micromyzus katoi* *sensu* Eastop 1966) by:

- longer siphunculi: 2.57–3.08 of cauda vs. less than 2.4 in *Micromyzus katoi*, *Micromyzus vandergooti*
- different ratio of VIa/VIb: apterae 3.87–4.29 (but alatae: 4.54–4.75) vs. 4.4–6.0 in *Micromyzus katoi*;
- higher ratio of siphunculus length/diameter of siphunculus in the middle: apterae: 8.17–8.98, alatae 7.04–8.86 vs. 5–8 in *Micromyzus katoi* *sensu* Eastop, 1966;
- lack of dorsal sclerotisation vs. dorsal sclerotic crossbars in *Micromyzus diervillae*, *Micromyzus mawphlangensis*;
- pale cauda vs. dark cauda in *Micromyzus niger*;
- pale tibiae vs. dark tibiae in *Micromyzus osmundae*, *Micromyzus nikkoensis*;
- higher number of accessory hairs on ultimate rostral segment than in *Micromyzus pojanii*;
- lower number of secondary rhinaria on antennal segment III than in *Micromyzus hangzhouensis*.

The following key may be applied, which is a modification of the last part of the key to apterae on *Polypodium* and other fern-feeding aphids from Blackman and Eastop (2006). Using their key, *Micromyzus platycerii* will run to couplet 52, where the siph./cauda ratio is discriminatory:

**Table:** 

52	SIPH 2.5–3.7 × cauda	**53**
–	SIPH 1.6–2.4 × cauda	**57**
53	SIPH tapering/cylindrical, or slightly swollen subapically, at least 10 × longer than their width at midlength	**54**
–	SIPH slightly swollen in middle (cigar-shaped), less than 10 × longer than their width at midlength	**56**
54	R IV+V 1.34–1.62 × HT II, and bearing 8–15 accessory hairs. Dorsum with extensive dark sclerotisation, not segmentally divided	***Micromyzella davalliae***
–	R IV+V 0.85–1.05 × HT II, with only 2–6 accessory hairs. Dorsum pale or with broad dusky sclerotic cross bands	**55**
55	R IV+V with 2 accessory hairs. Cauda much paler than SIPH, with several or all of hairs short and blunt	***Micromyzella sleonensis***
–	R IV+V with 5–6 accessory hairs. Cauda dusky/dark with all hairs fine-pointed	***Micromyzus pojanii***
56	R IV+V c.1.1 × HT II, with 4 accessory hairs. Dorsum mainly pale, with a fragmented spinal patch on ABD TERG 1–3	Micromyzus mawphlangensis*
–	R IV+V 1.67–2.25 × HT II, with 8–12 accessory hairs	**61**
57	HT II 0.104–0.118 mm long. R IV+V 1.05–1.24 × HT II. SIPH thin and cylindrical	**58**
–	HT II 0.07–0.09 mm long. R IV+V 1.25–2.3 × HT II. SIPH rather thick, often somewhat cigar-shaped	**59**
58	First tarsal segments all with 2 hairs. Cauda dark. ANT BASE VI 0.132–0.155 mm. R IV+V 0.81–0.92 × ANT BASE VI	***Micromyzella kathleenae***
–	First tarsal segments all with 3 hairs. Cauda pale. ANT BASE VI 0.099–0.127 mm. R IV+V 0.91–1.13 × ANT BASE VI	***Micromyzella sophiae***
59	R IV+V 1.7–2.3 × HT II and bearing 8–14 accessory hairs. ANT PT/BASE 4.4–6.0	***Micromyzus katoi* group**
–	R IV+V 1.2–1.7 × HT II and bearing 4–9 accessory hairs. ANT PT/BASE 2.5–4.6	**60**
60	SIPH 1.6–2.0 × cauda. ANT III without rhinaria (?). ANT PT/BASE 2.5–3.3. First tarsal segments with 3 or 4 hairs	Micromyzus vandergooti*
–	SIPH 2.0–2.3 × cauda. ANT III usually with 1 or more rhinaria. ANT PT/BASE 3.0–4.6. First tarsal segments with 2 or 3 hairs	***Micromyzella filicis***
61	Dorsum uniformly dark	***Micromyzella pterisoides***
–	Dorsum pale, membranous	***Micromyzus platycerii* sp. n.**

#### Descriptions.

**Figure 1.**
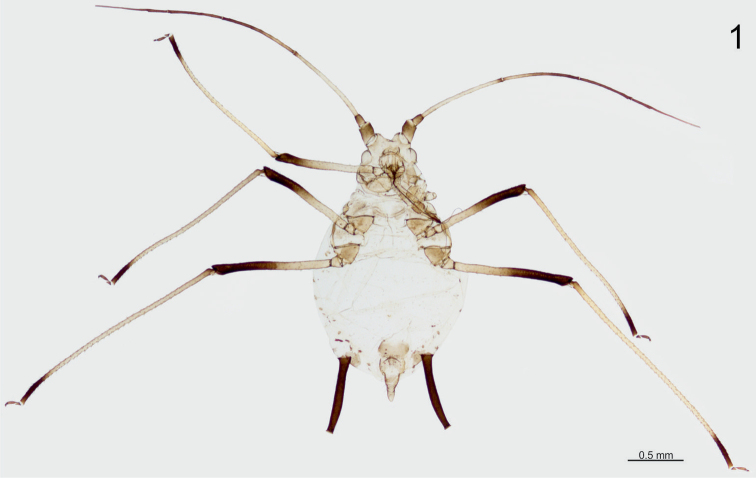
Microscopic slide of the holotype specimen – apterous viviparous female of *Micromyzus platycerii*.

**Figures 2–4.**
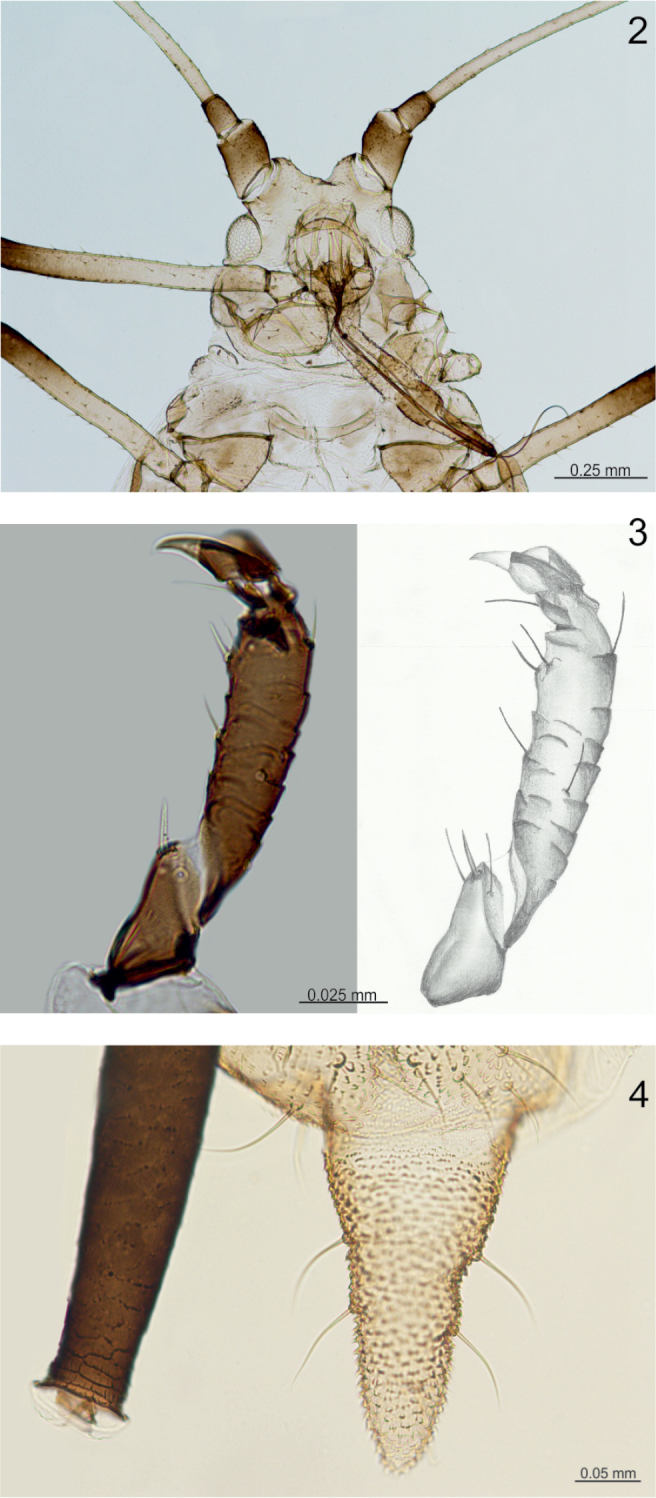
Morphological features of apterous viviparous female of *Micromyzus platycerii*: **2** head and rostrum **3** tarsus of hind leg **4** cauda and apex of siphunculus.

Apterous viviparous female (Fig. 1) (measurements based on 6 specimens). Body in life brown, with reddish eyes. Body 2.16–2.68 mm long (incl. cauda), weakly pigmented. Head weakly sclerotised, with sparse and minute spicules on well developed, diverging frontal tubercles (Fig. 2). Antenna 6 segmented, 2.96–3.23 mm long, 1.20–1.26 of body length, covered with short setae, shorter than basal diameter of antennal segment III. Antennal segments I and II dusky, antennal segment III pale, with darker tip and 1- 2 secondary rhinaria on basal part, 0.68–0.81 mm long; antennal segment IV darker towards the end, 0.61–0.72 mm long; antennal segment V dark, 0.42–0.51 mm long; antennal segment VI dark, VIa: 0.17–0.19 mm, VIb: 0.74–0.77 mm, ratio VIa/VIb 3.87–4.29. Rostrum 0.71–0.82 mm long, reaching to the hind coxae, 0.28–0.34 of body length, 0.91–1.16 of the length of antennal segment III. Ultimate rostral segment 0.186–0.192 mm long, 1.81–2.00 of the second segment of hind tarsus, with 8–11 accessory setae.

Prothorax sclerotised, mesothorax with marginal and spinal sclerites, metathorax with marginal sclerites only. Mesothoracic furca not separated, weakly pigmented. Legs covered by short, pointed hairs, shorter than the middle diameter of tibia. Distal half of femora dark; tibiae pale except for dark apices, tarsi black. Ventral side of each first tarsal segment with one thick, pointed, peg-like seta and two thinner setae (Fig. 3). Second segment of tarsus 0.096–0.103 mm long.

Abdomen membranous, with single, very small marginal scleroites, sometimes bearing a small marginal tubercle on abdominal tergite II or III. Small antesiphuncular and bigger postsiphuncular sclerites present. Each tergite with a row of short, pointed setae. Reniform spiracles placed at the posterior end of small scleroits. Siphunculi dark, slightly swollen in the middle, discretely imbricated, with 2–3 rows of distinct imbrications just below the apex (Fig. 4), 0.59–0.69 mm long, 2.57–2.77 of cauda. Subgenital fig broadly elliptical, 0.26–0.31 mm wide, with 2–3 longer setae on its anterior border and a row of shorter setae at its posterior border. Cauda pale, finger-shaped with broader base, 0.22–0.27 mm long, with 4–5 setae (Fig. 4).

**Figure 5.**
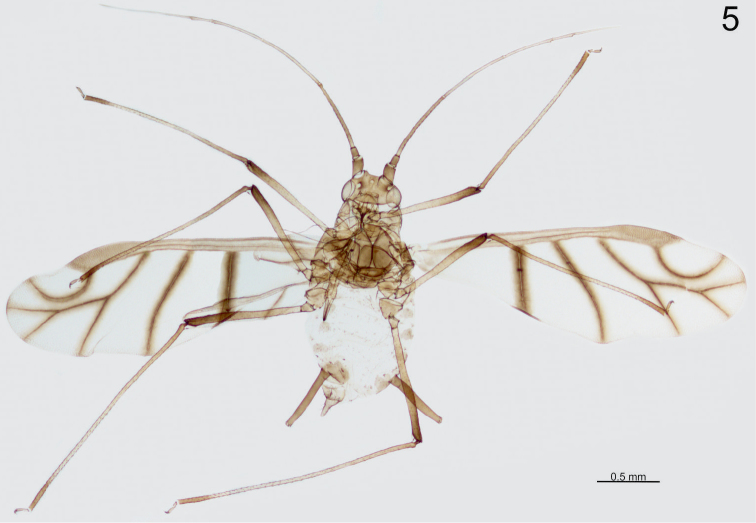
Microscopic slide of the paratype specimen – alate viviparous female of *Micromyzus platycerii*.

**Figures 6–8.**
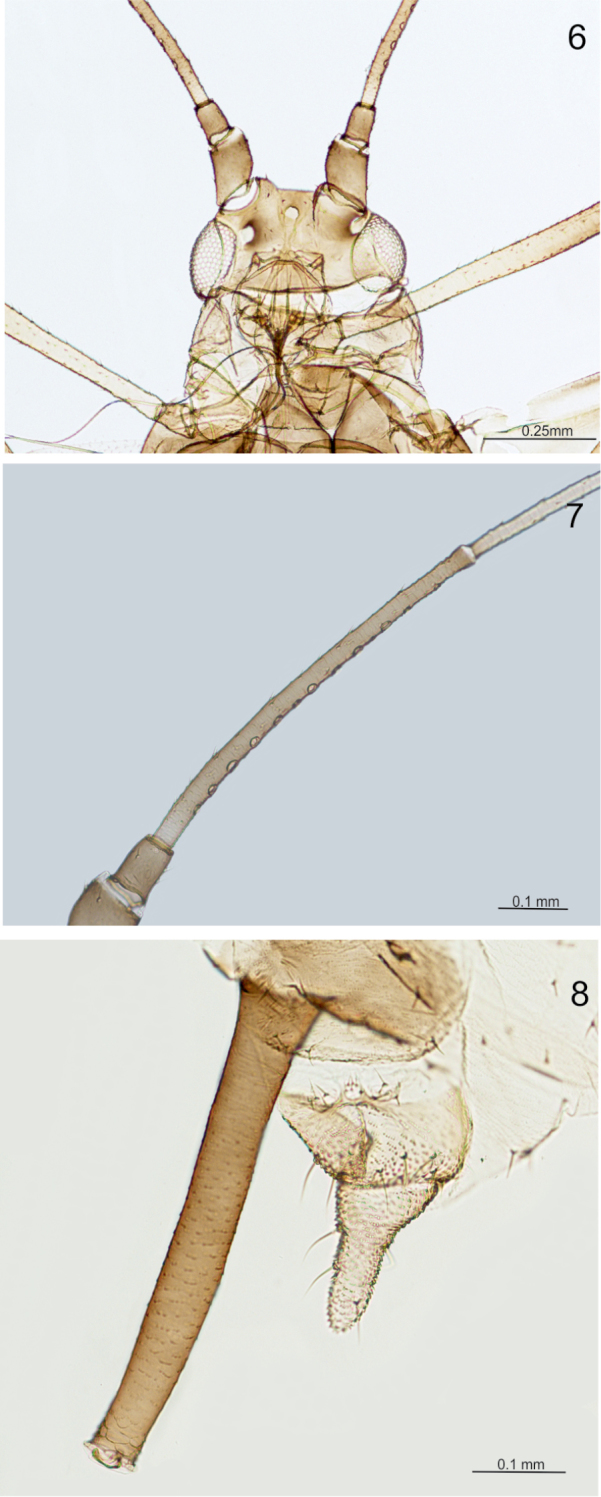
Morphological features of alate viviparous female of *Micromyzus platycerii*: **6** head **7** 3^rd^ antennal segment **8** cauda and apex of siphunculus.

Alate viviparous female (Fig. 5) (measurements based on 2 specimens). Body in life brown, 1.95–1.97 mm long (incl. cauda). Head sclerotised, dark and smooth, with low, divergent frontal tubercles, only delicately imbricated; covered sparsely with a few short, pointed setae (Fig. 6). Compound eyes well developed, with triommatidium. Antennae 2.63–2.69 mm long, 1.35–1.36 of body length; antennal segments I–III darker than IV–VI. Length of antennal segments: III 0.56–0.59, IV 0.55–0.56, V 0.42–0.43, VIa 0.15–0.17, VIb 0.73–0.76, ratio of VIb/VIa 4.54–4.75. Antennal segment III with 11–14 secondary rhinaria along its entire length (Fig. 7), segment covered with setae shorter than 0.5 of its basal diameter. Rostrum 0.85–0.90 mm long, 1.43–1.59 of the length of antennal segment III, 0.43–0.46 of body length, reaching past hind coxae. Ultimate rostral segment 0.18–0.19 mm long, 2.00–2.07 of the second segment of hind tarsus, with 8–9 accessory setae.

Thorax heavily sclerotised. Wings with very dark pigmentation of veins and their borders, especially on proximal cubital vein (Fig. 2). Media of fore wing with two forks. Radial sector strongly curved. Legs dusky, with darker apices of femora and tibiae, covered with short pointed setae. Tarsi dusky, first segment with 3 setae, including one sensory peg-like seta; second segment of hind tarsus 0.09 mm long.

Abdomen membranous, with transverse rows of short, pointed setae and with marginal sclerites only. Antesiphuncular and postsiphuncular sclerites well developed. Reniform spiracles placed at the posterior end of small scleroits. Genital fig broadly oval, 0.23–0.26 mm wide, with two setae on the anterior edge and 4–6 setae on the posterior edge. Siphunculi 0.50–0.51 mm long, 2.60–3.08 of the length of cauda, clavate, slightly swollen in the middle, dusky, with 2–3 rows of distinct imbrications at the apex, just below weakly developed flange (Fig. 8). Cauda finger-shaped, pale, 0.17–0.19 mm long, with 4–5 setae.

#### Biology.

The brown, shiny aphids were feeding on young shoots of *Platycerium coronarium*, in great numbers on the undersides of leaves.

### Taxonomic comments

*Micromyzus platycerii* sp. n. has 3 hairs on first tarsal segments, including one peg-like, sensory seta, which is a characteristic of the genus *Micromyzella* Eastop. The key presented by Su et al. (2014) leads to undeterminable point 9, with morphological features of apterae similar to *Micromyzella*, but of alatae similar to *Micromyzus*. However, species in the genus *Micromyzus*, including *Micromyzus katoi*, also have 3 setae on first tarsal segments (Noordam 2004, Miyazaki 1968). The generic classification of the collected specimens was primarily based on the morphology of wing, which in its pigmentation and strongly curved radial sector undoubtedly puts it into the genus *Micromyzus* (Blackman and Eastop 2006). (In a key to alate morphs by Noordam (2004) it is stated that the forewings of *Micromyzus katoi* are not bordered with black, but this is evidently a typographic error, and the forewing is correctly illustrated in the same work).

Viviparous females of *Micromyzus platycerii* sp n. are morphologically very similar to *Micromyzus katoi*. Both species have a membranous abdomen, dark siphunculi, clavate in shape and with a few rows of imbrications, pale cauda with 4–5 setae and darker tips of antennal segments (Takahashi 1925). They most resemble the Australian “*Micromyzus ? katoi*” of Eastop (1966), the only discriminant character being the ratio of length to middle diameter of siphunculi, which seems to be higher in the new species. From the illustration in Eastop‘s work, this ratio is lower (c. 5.75) in the alate morph of the Australian *Micromyzus katoi* than in alate of the new species (7.04–8.86). Furthermore, according to Blackman and Eastop’s (2006) key, apterae of *Micromyzus katoi* (including Eastop’s *Micromyzus katoi*) have a siphunculus/cauda ratio of 2.4 or less, whereas in apterae of the new species it is well above this value.

The observed differences, including the variable chaetotaxy of the first tarsal segments, put in question taxonomic relations within the *Micromyzus*/*Micromyzella* group and indicate a strong need for revision of the fern-feeding aphids of the tropical and subtropical region.

## Supplementary Material

XML Treatment for Micromyzus platycerii
